# First-in-Human Percutaneous Epicardial-Only Left Atrial Appendage Closure Using Sierra Left Atrial Appendage Ligation System

**DOI:** 10.3390/jcm13237417

**Published:** 2024-12-05

**Authors:** Jakub Batko, Radosław Litwinowicz, Boguslaw Kapelak, Krzysztof Bartuś

**Affiliations:** 1 Department of Anatomy, Jagiellonian University Medical College, 31-008 Krakow, Poland; 2 CAROL—Cardiothoracic Anatomy Research Operative Lab, Department of Cardiovascular Surgery and Transplantology, Institute of Cardiology, Jagiellonian University Medical College, 30-688 Krakow, Poland; 3 Department of Cardiovascular Surgery and Transplantology, Institute of Cardiology, Jagiellonian University Medical College, St. John Paul II Hospital, 31-202 Krakow, Poland

**Keywords:** left atrial appendage, left atrial appendage closure, Sierra device

## Abstract

**Background:** In patients with atrial fibrillation and contraindications for oral anticoagulation, in which an increased risk of stroke remains, a left atrial appendage exclusion should be considered for elimination, because the left atrial appendage is the most common site of thrombus. The aim of this study is to present the first-in-human study results of the Sierra Aegis Left Atrial Appendage Ligation System, a new epicardial-only left atrial appendage closure system. **Methods:** This study was a prospective, first-in-human, single-center study evaluating the effectiveness and safety of the Sierra Aegis Left Atrial Appendage Ligation System device for epicardial left atrial appendage closure. Seven patients (mean age: 57.3 ± 10.6 years, 71.4% male) were qualified for a left atrial appendage closure because of an increased risk of bleeding with the need for lifelong anticoagulation pharmacology due to an increased risk of stroke. The patients’ preoperative and intraoperative characteristics were collected. Patients were observed during their 1-month, 3-month, 6-month, and 1-year follow-up. **Results:** The mean procedure time was 21.2 ± 8.2 min. All patients spent 3 days in the hospital including monitoring, the performance of preoperative CT scans, and anatomical evaluation. No tamponade, bleeding, thrombus, or left atrial appendage leakage were observed during the procedure or in-hospital stay. During the 1-month, 3-month, 6-month, and 1-year follow-up visits, none of the patients reported any complications. No tamponade, leakage, or left atrial appendage thrombus were observed. **Conclusions:** This first-in-human study regarding Sierra use for left atrial appendage closure shows promising results regarding the effectiveness and safety of the Sierra device for use in humans.

## 1. Introduction

### 1.1. Atrial Fibrillation Epidemiology, Causes, and Treatment

Atrial fibrillation is the most common cardiac arrythmia, which leads to a decrease in life quality, decrease in exercise tolerance, and even fatal complications including stroke [1,2,3,4]. Its development can be connected to an underlying genetic predisposition, atrial fibrosis, or atrial enlargement [5,6]. Over 100 genetic loci predisposing to the development of atrial fibrillation have been described to date; these are associated with inflammation, electrical abnormalities associated with ion channels dysfunction, and structural remodeling. The first line of treatment includes pharmacological, antiarrhythmic treatment, or electrical cardioversion in certain conditions. In patients unresponsive to antiarrhythmic treatment, oral anticoagulation is indicated for the prevention of fatal thromboembolic complications [2,7,8]; however, there is a wide group of patients with contraindications for oral anticoagulation in whom an increased risk of stroke remains [9,10,11,12]. The main contraindications for anticoagulation treatment include the following: known hypersensitivity to the active ingredient or one of the excipients of the drug formulation, mechanical heart valve, rheumatic mitral stenosis, severe renal impairment, triple positive antiphospholipid syndrome, significant inherited or acquired bleeding disorder, clinically significant active bleeding, hepatic disease with associated coagulopathy, lesions or conditions with significant risk of bleeding (including intracranial hemorrhage), pregnancy, and breastfeeding. Despite this wide range of anticoagulation contraindications, only some of them are absolute lifelong contraindications.

### 1.2. Left Atrial Appendage Exclusion Indications

According to the most recent guidelines, in this group of patients a left atrial appendage exclusion should be considered to eliminate the most common site of thrombus (the left atrial appendage) [2,13,14]. As per the most recent AHA guidelines, in patients with an atrial fibrillation and an increased risk of stroke, which can be evaluated by CHA2DS2-VASc score over 2 and an irreversible contraindication to oral anticoagulation, a percutaneous left atrial appendage occlusion is reasonable [15]. Additionally, these guidelines mention that it can be a reasonable alternative, based on patient preference, after careful consideration. For patients with a diagnosed atrial fibrillation undergoing cardiac surgery and an increased risk of stroke, a surgical left atrial appendage closure is recommended with the use of anticoagulation [15]. A left atrial appendage closure is especially beneficial in elderly patients and individuals with renal dysfunction [16,17].

### 1.3. The Left Atrial Appendage Exclusion Devices

The left atrial appendage exclusion devices can be divided into two main types: epicardial and endocardial devices. Epicardial devices include the AtriClip (AtriClip PRO Device, AtriCure, Dayton, OH, USA) and the Lariat device. The AtriClip device, designed for use during cardiac surgery, consists of a clip located on a deploying device. It simplifies the left atrial appendage exclusion process, which saves time during complicated cardiac surgery with results comparable to surgical suturing and excision of the appendage. In some cases, it can be used through a mini-thoracotomy as an isolated procedure. The Lariat device, which requires both vascular and epicardial access, consists of a suture loop and guide wire, which is located within the left atrial appendage lumen. It provides for a complete isolation of the left atrial appendage without the presence of a foreign body within the patient’s cardiovascular system and pericardium [18,19]. Endocardial devices include the Watchman (Boston Scientific, Marlborough, MA, USA), the Amulet (Abbott, Abbott Park, IL, USA), the LAmbre (Lifetech Scientific Corp., Shenzhen, China), and the WaveCrest (Biosense Webster, Irvine, CA, USA), which all require vascular percutaneous endocardial access [20,21,22,23,24]. The Watchman, and more precisely its newest version (the Watchman FLX), is the first and one of the most commonly implanted left atrial appendage occlusion devices; it is deployed within the left atrial appendage neck and anchored to its wall. The Amulet and LAmbre devices are next-generation occlusion devices which additionally implement a disc; despite the occluder being located within the neck, it can tightly adhere to the wall of the left atrium around the left atrial appendage orifice. Each device has specific requirements and limitations when they are used. Because of this and a common consensus that no ideal left atrial appendage closure device is yet available on the market, further development of new devices is needed.

### 1.4. The Left Atrial Appendage Clinical Anatomy

The anatomy of the left atrial appendage is one of the most crucial components for successful left atrial appendage closure [25,26,27,28,29,30,31,32]. The left atrial appendage can be divided into two parts, as follows: the neck and the lobe [33]. Within the left atrial appendage neck, the landing zone for most of the occlusion devices can be found [17,34]. In most recent studies, malformations of the left atrial appendage neck, which is crucial for a safe left atrial appendage closure, have been described [35]. Detailed left atrial appendage anatomy limits the use of different left atrial appendage closure and occlusion devices and is associated with certain device-specific complications [36,37,38,39,40,41,42,43].

There is a lack of devices which can provide a percutaneous, epicardial-only, and subxiphoid approach, and which do not leave foreign bodies inside the patient’s heart, and can be used as a stand-alone short-duration procedure in a Cath Lab.

### 1.5. The Aim of the Study

The aim of this study is to present the first-in-human results of the Sierra Aegis Left Atrial Appendage Ligation System, a new percutaneous, epicardial-only left atrial appendage closure system.

## 2. Materials and Methods

### 2.1. Study Design

This was a prospective, first-in-human, single-center study evaluating the effectiveness and safety of the Sierra Aegis Left Atrial Appendage Ligation System device (Aegis Medical Innovations Inc., Vancouver, BC, Canada) for epicardial left atrial appendage closure. Seven patients (mean age: 57.3 ± 10.6 years, 71.4% male) were qualified for a left atrial appendage closure due to increased risk of bleeding, with the need for lifelong anticoagulation pharmacology due to an increased risk of stroke. The patients’ preoperative and intraoperative characteristics were collected. The procedural time was measured as the skin-to-skin time. The patients were observed at their 1, 3, 6, and 12-month follow-up. During each follow-up visit, a transesophageal echocardiography was performed to confirm closure and an absence of leaks. The study was conducted in accordance with the Declaration of Helsinki (as revised in 2013) and was approved by the local Ethical Committee (nr 1072.61201.16.2012). Informed consent was collected from each study participant.

### 2.2. Device Description

**Figure 1:**
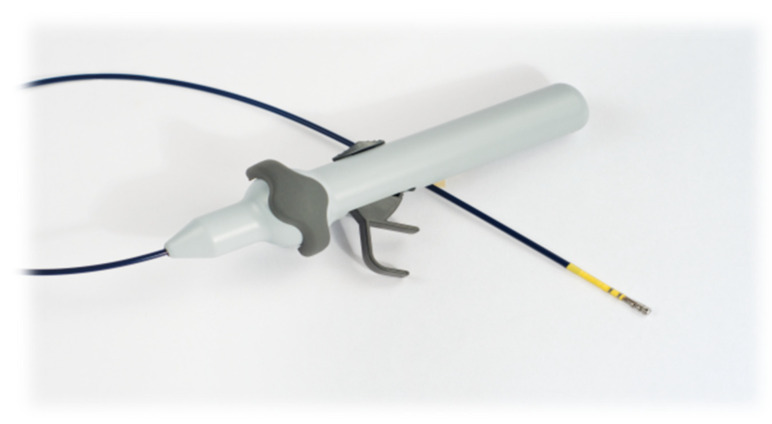
The Sierra Aegis Left Atrial Appendage Ligation System device. It consists of three parts (the introducer kit, the epicardial guiding device, and the ligating system). The introducer kit is connected to the handle, with a connector for an electrophysiology extension cable, and an epicardial guiding device finished with stabilization arms with electrodes.

The Sierra Aegis Left Atrial Appendage Ligation System is a device designed for ligation of the left atrial appendage epicardially, which uses a purely percutaneous approach without entering the bloodstream or heart chambers at any time. It consists of three parts: the introducer kit, the epicardial guiding device, and the ligating system (Figure 1).

**Figure 2:**
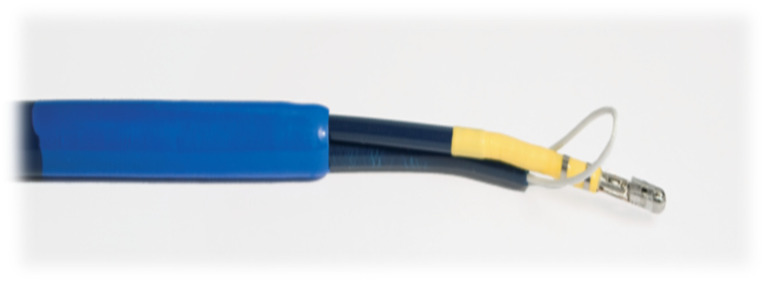
Sierra Aegis Left Atrial Appendage Ligation System device. Stabilization arms with visible suture loop delivery system. The suture loop is hollow and supported by nitinol wire (removable after suture closure) to provide rigidity and make it fluoroscopically visible during delivery.

The introducer kit includes a steerable introducer sheath and a dilatator. The epicardial guiding device is a flexible, steerable device finished with stabilization arms distally, with electrodes located on the arms which can measure the electric potential from the tissue (ECG). The stabilization arms can be opened and closed with the handle, which additionally has a locking mechanism for fixing the position of the arms. Additionally, its handle is equipped with a connector for an electrophysiology extension cable (Figure 2).

**Figure 3:**
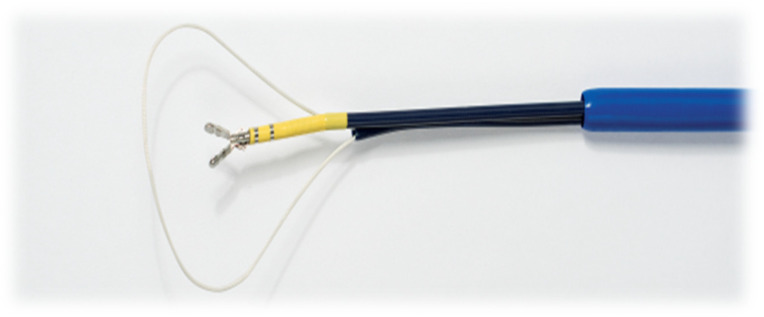
Sierra Aegis Left Atrial Appendage Ligation System device. Suture loop with suture cutter.

The ligating system includes a suture loop delivery system with a polyethylene non-absorbable, braided suture loop, and titanium suture lock. The delivery system has an outer sheath which the suture loop can both be drawn into or extended out of, in order to change its effective size. This allows the loop to be repositioned as necessary, pre-tightened, and assessed before delivering the suture lock. In effect, this provides a one size fits all solution. The suture loop is hollow and supported by nitinol wire (removable after the suture closure) to provide rigidity and make it fluoroscopically visible during delivery. The suture tails are trimmable with the supplied flexible suture cutter (Figure 3).

### 2.3. Procedure Description

**Figure 4:**
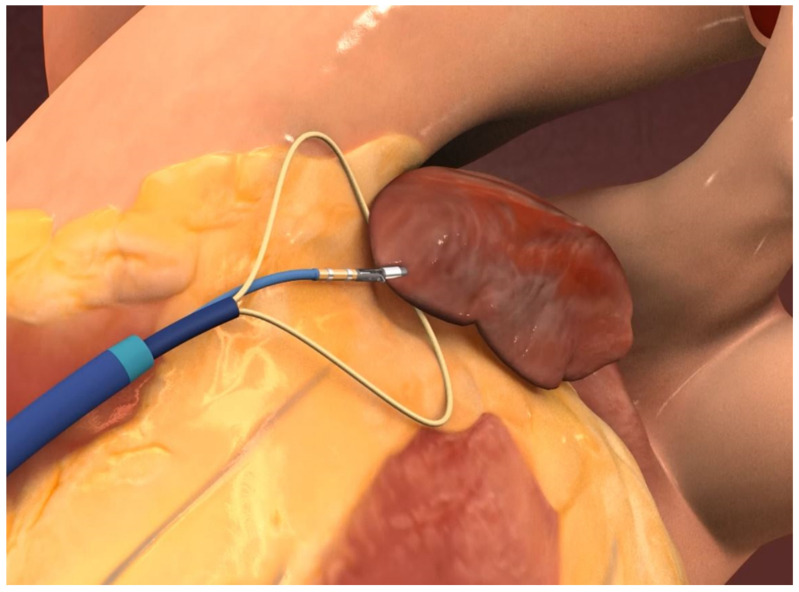
Sierra Aegis Left Atrial Appendage Ligation System device procedure visualization. After confirmation of the left atrial appendage tissue capture, the locking mechanism is engaged to sustain the arms’ position. The loop size is adjusted to fit the desired size of the left atrial appendage. The position of the device can be changed as many times as is needed for optimal left atrial appendage exclusion.

The Sierra Aegis Left Atrial Appendage Ligation System device requires a percutaneous subxiphoid approach for epicardial access. An appropriate electrophysiology cable is attached to the epicardial guide. To record the electrograms, the lead pins are connected to the amplifier. The suture loop is preloaded over the guiding device. The guiding device, with the arms of the guiding device closed, is inserted through the introducer under fluoroscopic guidance. The distal arms are then opened and closed to capture the desired tissue, and based on the electrograms displayed from the arms, the tissue characteristics are captured. After confirmation of the left atrial appendage tissue capture, the locking mechanism is engaged to sustain the arms’ position. The ligation system is introduced and advanced through the introducer (Figure 4).

The ligating system is introduced through the introducer and the loop is advanced to the site of ligation under fluoroscopic guidance. The loop size is adjusted to fit the desired size of the left atrial appendage. After loop positioning and pre-tightening based upon the operator’s desired position, the outer sheath is locked into position. The position of the device can be changed as many times as is needed for optimal left atrial appendage exclusion. The ligature is permanently secured around the tissue by removing the wire from the suture and advancing the suture lock forward. After closure verification, the delivery system and the guiding device are removed, and the ligature tails (extending out of the proximal end of the sheath) are trimmed with a suture cutter. There is no need for periprocedural anticoagulation use. For all the procedures, local anesthesia of the incision region with lignocaine solution and mild sedation with midazolam are used.

During the procedure, as during any other left atrial appendage occlusion procedure, transesophageal echocardiography (Philips IE 33, Philips, Warsaw, Poland) is performed to ensure correct left atrial appendage exclusion and for verification there is no leakage.

### 2.4. Statistical Analysis

The data were analyzed using IBM SPSS Statistics 29.0 (Predictive Solutions, Pittsburgh, PA, USA). Categorical variables are presented as numbers (*n*) or percentages. Quantitative variables are presented as mean with standard deviation.

## 3. Results

### 3.1. Patients’ Characteristics

Seven patients with contraindications for anticoagulation treatment (mean age: 57.3 ± 10.6 years, 71.4% male) underwent a left atrial appendage exclusion with the Sierra Aegis Left Atrial Appendage Ligation System device. Three patients had diabetes mellitus type 2 and heart failure. All the included patients were overweight, and three of them were obese. Two patients had paroxysmal atrial fibrillation. The patients’ contraindications to oral anticoagulation included labile international normalized ratio, frequent falls, gastrointestinal bleeding, intracranial hematoma, and hemorrhagic diathesis. The detailed characteristics of each patient are provided in Table 1.

### 3.2. Procedure and Hospital Stay Details

The mean procedure time was 21.2 ± 8.2 min. All the patients spent 3 days in the hospital. No tamponade, bleeding, thrombus, or left atrial appendage leakage over 1 mm were observed during the procedure or the in-hospital stay.

### 3.3. Postprocedural Follow Up

During the 1-month, 3-month, 6-month, and 1-year follow-up visit, none of the patients reported any complications. No tamponade, leakage, or left atrial appendage thrombus were observed in the transesophageal echocardiography up to 1 year postprocedure.

## 4. Discussion

### 4.1. Results Discussion

This pilot study, involving a small population of seven patients, shows promising results of left atrial appendage closure using the Sierra Aegis Left Atrial Appendage Ligation System. The procedure time was comparatively brief and required a single operator, epicardial-only, and purely percutaneous approach. The patients’ hospital stay was 3 days due to the diagnostic CT needed, the novelty of the device, and for observation of short-term complications; however, it could be shortened to a single day. Despite some common difficulties experienced with the first-in-human use of this device, and no prior experience with using the Sierra Aegis Left Atrial Appendage Ligation System device, three cases of left atrial appendage closure in obese patients were performed with a procedure time under 15 min, and with successful, uncomplicated left atrial appendage ligation. The heterogeneity of the group should be mentioned (three patients had diabetes mellitus type 2 and heart failure). All of the included patients were overweight, and three of them were obese. Two patients had paroxysmal atrial fibrillation. The patients’ contraindications to oral anticoagulation included labile international normalized ratio, frequent falls, gastrointestinal bleeding, intracranial hematoma, and hemorrhagic diathesis. As mentioned above, these promising results suggest that future randomized trials regarding the use of the Aegis Left Atrial Appendage Ligation System may be beneficial in providing an additional epicardial left atrial appendage closure device with a satisfactory safety profile.

### 4.2. Left Atrial Appendage Exclusion Devices Comparison

To understand the need for a new, percutaneous epicardial-only left atrial appendage closure device that does not require entering the bloodstream or heart chambers, other available devices should be discussed. The most commonly used endocardial left atrial appendage occlusion device is the Watchman or Watchman FLX [44,45,46]. It requires an endocardial approach, leaves an implant inside the patient’s body, and requires two physicians to perform the procedure. The mean procedure time is one hour. To perform the procedure, a bloodstream entrance, transseptal puncture, full heparinization, contrast use, and postoperative anticoagulants are needed. The most common complications are thrombosis, thrombus formation, device embolization, and device-related stroke [47]. To address some of these concerns, additional devices have been developed including the Amulet and the LAmbre; these led to a decrease in the rates of thrombosis, thrombus formation, and other complications [47]. For further development, and to avoid the postprocedural use of anticoagulation, the Lariat device was invented [18,48]. It still requires vascular access; however, it does not leave an implant inside the patient’s body due to epicardial closure with the loop. The additional benefits of the Lariat devices are as follows: the elimination of device-related thrombosis and thrombus formation, less potential for device migration and electrical isolation of the left atrial appendage, a reduction in blood pressure, and the impact on systemic homeostasis [48,49]. The Sierra Aegis Left Atrial Appendage Ligation System is a next-generation device which requires only one epicardial percutaneous subxiphoid access. Only one physician is needed for the procedure. The procedure time can be under 15 min, it does not require vascular access or heart chamber access, and there is no need for any blood contrast injection. It is provided as a one size- fits all device with the ability to redo the procedure. Additionally, it does not require heparinization of the patient during the procedure or the use of anticoagulation drugs after the procedure. Such characteristics, which indicate the potential usefulness of the Sierra Aegis Left Atrial Appendage Ligation System device in certain clinical situations, are especially significant when the main group of patients in need is considered. Its zero contrast requirement may be a great fit for patients with renal impairment or for elderly patients with multiple comorbidities. Its short procedural time without the need for heparinization and general anesthesia makes it one of the best choices in high and very high risk patients, who are often perfect candidates for left atrial appendage exclusion due to the large number of medications taken (which commonly interact with oral anticoagulative drugs). This minimally invasive procedure, which does not require postprocedural lifetime anticoagulation, may be a very good choice for patients with a contraindication for anticoagulation and patients taking complex pharmacological therapies who are prone to unfavorable drug interactions. Also, this device is a great choice for the treatment of patients with anatomical contraindications for endocardial closure. Its one size fits all design and potential adaptation does not make this procedure left atrial appendage size-dependent. However, some types of left atrial appendage malformations may make it unfavorable, despite its size adaptation [35]. It is unknown how concomitant procedures may impact the safety of the Sierra Aegis Left Atrial Appendage Ligation System device. Several studies were performed to evaluate such a topic in other devices, as described above [20,50].

### 4.3. Procedural Time, Safety Profile, and Long-Term Outcomes Comparison

Comparing the Sierra Aegis Left Atrial Appendage Ligation System device with the Watchman FLX, based on previously published studies, it has a shorter procedural time (21.2 ± 8.2 vs. 44.8 ± 20.7 min), comparable due to the low number of Sierra Aegis Left Atrial Appendage Ligation System device studies performed, and periprocedural (0% vs. 1.8%) and follow-up (0% vs. 5.4%) complications [51,52]. Most of the Watchman FLX complications were associated with device-related thrombus (4.7%), which cannot be observed for the Sierra Aegis Left Atrial Appendage Ligation System device, due to its characteristics.

Comparing the Sierra Aegis Left Atrial Appendage Ligation System device with the LAmbre device, based on a previously published study with the best reported procedural time, it has a shorter procedural time (21.2 ± 8.2 vs. 29.0 ± 10.1 min), comparable due to the low number of Sierra Aegis Left Atrial Appendage Ligation System device studies performed, and periprocedural (0% vs. 0%) and follow-up (0% vs. 0%) complications [53].

Comparing the Sierra Aegis Left Atrial Appendage Ligation System device with the Amplatzer Amulet device, based on previously published studies, it has a shorter procedural time (21.2 ± 8.2 vs. 50.8  ±  18.4 min), comparable due to low number of Sierra Aegis Left Atrial Appendage Ligation System device studies performed, and periprocedural (0% vs. 1.5%) and follow-up (0% vs. 2.2%) complications [54].

Comparing the Sierra Aegis Left Atrial Appendage Ligation System device with the Lariat device, based on previously published studies, it has a shorter procedural time (21.2 ± 8.2 vs. 45 (36–55) min), comparable due to low number of Sierra Aegis Left Atrial Appendage Ligation System device studies performed, and periprocedural (0% vs. 3.3%) and follow-up (0% vs. 5%) complications [41,48].

It should be noted that detailed comparisons of the devices should be performed in randomized controlled trials, despite these initial promising results.

### 4.4. Device Contraindications

Some of the device limitations should be mentioned. The device cannot be used in patients with a history of previous procedures requiring access to the pericardial space, including cardiac surgery. Other contraindicated patients may be identified in similar scenarios to any other left atrial appendage occlusion device, which would include patients with clots located within the left atrial appendage or left atrium during screening.

### 4.5. Left Atrial Appendage Anatomy Impact on Exclusion Procedures

#### 4.5.1. The Left Atrial Appendage Clinical Anatomy

The left atrial appendage is a diverticulum of the left atrium with a complicated structure and clinical impact [25,55,56,57]. It consists of two main parts (the neck, and the lobe) [33]. The left atrial appendage neck, described recently, borders clinically significant structures, including the left circumflex artery and pulmonary veins [26,27]. It can be divided into four surfaces (the aortic, arterial, free, and venous) which are identified based on their adjacent structures. The aortic and venous surfaces are identified as the thinnest; based on this parameter, they are the most prone to laceration during cardiovascular interventions [35]. Several classifications of the left atrial appendage have been developed, including the most recent, three-type classification [58]. It divides the left atrial appendages based on visual assessment into the arrowhead, cauliflower, and chicken wing, which provides a nomenclature for communication between professionals. Its shape additionally impacts adjacent structures, especially the cardiac transverse and oblique sinuses, important in epicardial left atrial appendage exclusion and surgical ablation procedures [59,60,61].

#### 4.5.2. The Left Atrial Appendage Malformations Impact Exclusion Device Choice

Recently, left atrial appendage malformations were described [35]. The two main types were identified as bulges and spikes. Bulges, defined as semi-curved epicardial projections with a shallow lumen and a large endocardial orifice, are theoretically associated with leakage of occluding devices inserted into the left atrial appendage neck. Spikes, described as elongated epicardial protrusions with a deep lumen and a small orifice, are theoretically anatomical contraindications for loop-based devices, which includes both the Lariat device and the Sierra Aegis Left Atrial Appendage Ligation System device.

#### 4.5.3. Left Atrial Appendage Anatomy in Patients with Atrial Fibrillation

The left atrial appendage anatomy in patients with atrial fibrillation differs from the one observed in healthy individuals [32,62,63,64]. Atrial fibrillation leads to an increase in orifice diameters (both anteroposterior and transverse) and orifice area and perimeter. Additionally, a left atrial appendage orifice oval shape was most commonly seen in patients with atrial fibrillation. The lobe part of the left atrial appendage does not differ between healthy individuals and patients with atrial fibrillation. It is worth noting that the left atrial appendage ejection fraction is significantly lower in patients with atrial fibrillation. To avoid complications associated with the complex anatomy of the left atrial appendage, new techniques of imaging are being developed and implemented, including three-dimensional intracardiac echocardiography [65].

### 4.6. Left Atrial Appendage Exclusion Impact on Systemic Homeostasis

The left atrial appendage is responsible for cardiac arrythmia in a number of patients, which was proven based on an analysis of patients with recurrent arrythmias [25]. Its anatomy, observed preoperatively, indicates postoperative homeostatic changes in patients [66,67]. Preoperative enlargement of the left atrial appendage is associated with unfavorable coagulation changes when compared with healthy individuals. The left atrial appendage exclusion impact on systemic homeostasis was mainly described in patients undergoing left atrial appendage exclusion with the Lariat system [67]. It can be suspected that due to similar left atrial appendage exclusion mechanisms, the same results can be achieved with the Sierra Aegis Left Atrial Appendage Ligation System device. The left atrial appendage exclusion led to adrenaline, noradrenaline, and aldosterone decreases in the short-term (at the 3-month follow-up). It is worth noting that endocardial occluder devices, implanted into the lumen of the left atrial appendage neck, did not induce such changes in patients who underwent such procedures. Additionally, a significant increase in adiponectin and insulin was observed in patients with left atrial appendage excluded with the Lariat device [67]. N-terminal pro-A-type natriuretic peptide and N-terminal pro–B-type natriuretic peptide significantly decreased shortly after epicardial LAA device implantation; however, no substantial changes were seen in baseline comparison with the 3-month follow-up [67]. A left atrial appendage exclusion with the Lariat device has an additional positive impact on left atrium modeling. It leads to an improvement in reservoir function of the left atrium. Additionally, conduit function and left atrium volume index improves after left atrial exclusion [49]. The left atrial appendage also serves as reservoir of stem cells, which may be used for the treatment of cardiac tissue scars after myocardial infarction [68,69]. However, it should be noted that the potential harvest of stem cells may be complicated during percutaneous, minimally invasive left atrial appendage exclusion procedures; a potential further adaptation of the Sierra Aegis Left Atrial Appendage Ligation System device may be considered to serve this purpose.

## 5. Conclusions

This first-in-human study of the Sierra Aegis Left Atrial Appendage Ligation System device for left atrial appendage closure shows very promising results regarding the effectiveness and safety of this device use in humans. The Sierra Aegis Left Atrial Appendage Ligation System is a next-generation device, which requires only one epicardial percutaneous subxiphoid access point. Only one physician is needed for the procedure. The procedure length can be under 15 min and it does not require vascular access or heart chamber access, and there is no need for blood contrast injection. It is provided as a one size fits all device, with the ability to redo the procedure. Additionally, it does not require heparinization of the patient during the procedure or the use of anticoagulation drugs after the procedure.

## Figures and Tables

**Table 1:** Patient characteristics. OAC: oral anticoagulants, GI: gastrointestinal, INR: international normalized ratio.

Consecutive Patient Number	1	2	3	4	5	6	7
Sex	M	M	M	F	M	F	M
Age	59	61	48	74	47	68	44
OAC contraindication	GI bleeding	Intracranial hematoma	Hemorrhagic diathesis	Frequent falls	Labile INR	GI bleeding	Labile INR
Body Mass Index (kg/m^2^)	25	36	26	35	29	31	28
Diabetes type 2	yes	no	no	No	yes	yes	no
Heart Failure	no	yes	yes	Yes	no	yes	no
Stroke	no	no	yes	No	no	no	yes
Hypertension	yes	yes	no	Yes	yes	yes	no
Atrial fibrillation type	paroxysmal	persistent	paroxysmal	persistent	persistent	persistent	persistent
Ejection fraction (%)	65	50	45	45	60	50	60

## Data Availability

All available data are presented in the manuscript.
